# The Role of Inflammasomes in Glomerulonephritis

**DOI:** 10.3390/ijms23084208

**Published:** 2022-04-11

**Authors:** Paula Anton-Pampols, Clara Diaz-Requena, Laura Martinez-Valenzuela, Francisco Gomez-Preciado, Xavier Fulladosa, Anna Vidal-Alabro, Joan Torras, Núria Lloberas, Juliana Draibe

**Affiliations:** 1 Nephrology Department, Bellvitge University Hospital, Hospitalet de Llobregat, 08907 Barcelona, Spain; panton@bellvitgehospital.cat (P.A.-P.); lmartinezv@bellvitgehospital.cat (L.M.-V.); fgomezp@bellvitgehospital.cat (F.G.-P.); xfulladosa@bellvitgehospital.cat (X.F.); jbordignon@bellvitgehospital.cat (J.D.); 2 IDIBELL Biomedical Research Institute, Hospitalet de Llobregat, 08907 Barcelona, Spain; cdiazr@idibell.cat (C.D.-R.); avidala@idibell.cat (A.V.-A.); nlloberas@ub.edu (N.L.); 3 Clinical Sciences Department, Campus de Bellvitge, Barcelona University, Hospitalet de Llobregat, 08907 Barcelona, Spain; 4 Department of Physiological Sciences, Campus de Bellvitge, Barcelona University, Hospitalet de Llobregat, 08907 Barcelona, Spain

**Keywords:** inflammasome, NLRP3, glomerulonephritis, innate immunity

## Abstract

The inflammasome is an immune multiprotein complex that activates pro-caspase 1 in response to inflammation-inducing stimuli and it leads to IL-1β and IL-18 proinflammatory cytokine production. NLRP1 and NLRP3 inflammasomes are the best characterized and they have been related to several autoimmune diseases. It is well known that the kidney expresses inflammasome genes, which can influence the development of some glomerulonephritis, such as lupus nephritis, ANCA glomerulonephritis, IgA nephropathy and anti-GBM nephropathy. Polymorphisms of these genes have also been described to play a role in autoimmune and kidney diseases. In this review, we describe the main characteristics, activation mechanisms, regulation and functions of the different inflammasomes. Moreover, we discuss the latest findings about the role of the inflammasome in several glomerulonephritis from three different points of view: in vitro, animal and human studies.

## 1. The Inflammasome

**Figure 1:**
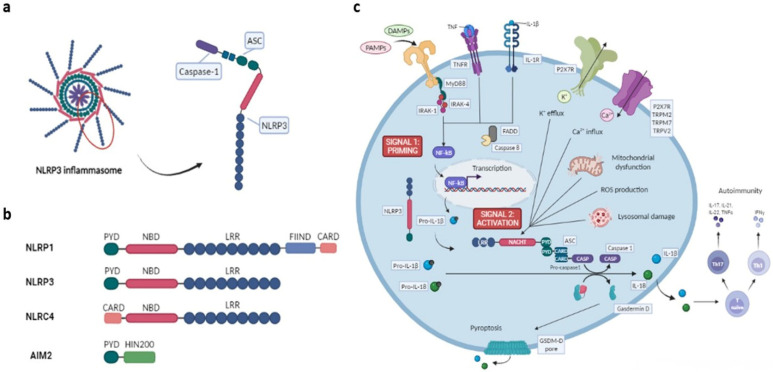
Inflammasome structure and mechanism of activation. (**a**) Schematic representation of NLRP3 inflammasome assembly and detailed conformation of NLRP3 scaffold, an adaptor apoptosis speck-like protein (ASC) and the effector procaspase-1. (**b**) Structure of NLRP1, NLRP3, NLRC4 and AIM2 which participate in the formation of the main inflammasomes. NLR family members (NLRP1, NLRP3, NLRC4) contain leucine-rich repeats (LRR) and central nucleotide binding domain (NBD). The N-terminal PYD domain is present in NLRP subfamily members, whereas NLRC4 presents a CARD domain. NLRP1 also contains a C-terminal extension containing a function-to-find domain (FIIND) and a CARD domain. AIM2 is composed of a N-terminal PYD domain and a C-terminal HIN (hematopoietic, interferon-inducible and nuclear localization) domain. (**c**) NLRP3 activation pathways and effector functions. NLRP3 inflammasome assembly can be triggered by several ways: PAMPs and DAMPs detection via PRRs, by cytokine stimulation via IL-1 receptor (IL-1R) or through TNF link to tumor necrosis factor (TNF) receptors TNFR1 and TNFR2. These elements trigger the transcription of NF-κB, which promotes the transcription of NLRP3 and IL1B genes; this is the first signal or priming. The second signal or activation can be produced by ionic flux, K^+^ efflux, Ca2^+^ influx, Na^+^ influx and Cl^-^ efflux, reactive oxygen species (ROS) and mitochondrial dysfunction or lysosomal damage. NLRP3 inflammasome assembly provokes IL-1β and IL-18 cytokines’ proteolytic maturation, which also participate in autoimmunity development and pyroptosis by the action of gasdermin-D. Protein myeloid differentiation primary response 88, MyD88; apoptosis signal-regulating kinase, ASK; kinases interleukin 1 receptor-associated kinase, IRAK; caspase-8, CASP8; Fas-associated protein with death domain, FADD; P2X purinoceptor 7, P2X7R; transient receptor potential melastatin, TRPM; transient receptor potential vanilloid, TRPV. Figure 1 has been created with BioRender.com.

The immune system is composed of two arms, the innate and adaptive immunity, that are responsible for both immediate and long-term immunity to pathogen- and non-pathogen-derived antigens. Innate immunity detects infections, changes in cellular homeostasis and tissue damage, subsequently generating inflammation, tissue repair and homeostatic balance restoration [1]. These effects are promoted by the recognition of pathogen-associated molecular patterns (PAMPs) and damage-associated molecular patterns (DAMPs). PAMPs and DAMPs bind to pattern recognition receptors, which include Toll-like receptors (TLRs), cytoplasmic NOD-like receptors (NLRs) and absent in melanoma 2-like receptors (AIM2) [2]. Previous studies have demonstrated the role of several members of the NLR family in the formation of inflammasomes, multiprotein complexes capable of recognizing inflammation-inducing stimuli. These complexes activate pro-caspase-1, which is responsible for the cleavage of multiple substrates, mainly the proinflammatory cytokines IL-1β and IL-18 [3]. The release of these cytokines by the inflammasome can also be carried out through an inflammatory form of programmed cell death named pyroptosis [4]. Therefore, the activation of the inflammasome develops innate immunity activity in response to tissue infection. Noninfectious stimulus can also activate the inflammasome [5]. Although inflammasomes can be activated by many members of the NLR family, this review will focus mainly on the NLRP3 inflammasome and NLRP1, NLRC4 and proteins absent in melanoma 2 (AIM2) (Figure 1a), also important in many immune diseases [6]. Furthermore, the role of these complexes in different glomerulonephritis will be reviewed.

## 2. NLR Family Inflammasomes

The NLR family comprises 23 human genes. Members of this family show common structural elements: C-terminal series of leucine-rich repeats (LRRs) and central nucleotide binding domains (NBD), a component of the larger NACHT domain [7,8]. Furthermore, NLR family members can be divided into different subfamilies depending on their N-terminal effector domain: caspase-activation and recruitment domain (CARD), baculovirus inhibitor of apoptosis protein repeat (BIR) or pyrin domain (PYD). The NLRP and NLRC subfamilies are the most important, the former being the best-characterized subfamily of NLRs. The NLRP subfamily members have PYD domains at their N-terminal while the NLRC proteins have one or more CARD domains [8,9,10]. NLR family members NLRP1, NLRP3 and NLRC4 have been the best studied in inflammasome formation [11].

### 2.1. NLRP Subfamily

The NLRP subfamily is composed of 14 members in human genome, plus 3 paralogs in mouse being NLRP1 (NALP1/CARD7) the first to be described in forming inflammasomes [12]. Its structure consists of a N-terminal PYD followed by a NACHT domain and LRRs. This is also contributed by a C-terminal extension containing a function-to-find domain (FIIND), which auto processes NLRP1 into two polypeptide chains, and a CARD domain, that leads to caspase-1 activation and the consequent proinflammatory cytokine release [13,14]. It has been reported that NLRP1 mutations can play a role in inflammatory diseases such as psoriasis [15], rheumatoid arthritis (RA) [16] or in systemic lupus erythematosus (SLE) [17].

NLRP3 inflammasome (Cryopyrin/Nalp3/Cias1/Pypaf1) is the most widely studied and is the only known member to be activated by numerous pathogenic and sterile inflammatory signals. Furthermore, NLRP3 plays a role in the regulation of IL-1β production in macrophages [18,19]. NLRP3 is composed of the NLRP3 scaffold, an adaptor apoptosis speck-like protein (ASC) and the effector procaspase-1. It interacts with ASC via PYD-PYD homotypic interactions to promote the formation of the inflammasome by recruiting and activating procaspase-1 to generate active caspase-1 (Figure 1b). This effector protein leads the conversion of the cytokine precursors pro-IL-1β and pro-IL-18 into mature and biologically active IL-1β and IL-18 [9,20]. The main attention given to the NLRP3 inflammasome has been due especially to its implication in the pathogenesis of several human inflammatory diseases, particularly of the cryopyrin-associated periodic syndromes (CAPS) [21]. Focusing on its critical role in regulating inflammation, the NLRP3 inflammasome could be of great importance to therapies targeting inflammation [22].

### 2.2. IPAF-NAIP Subfamily

Its most well-studied element, NLRC4 (IPAF/CARD12), was previously characterized as an ICE-protease activating factor (IPAF) regarding its capacity for activating caspase-1. Nevertheless, posterior studies clearly placed its domain structure in the NLR family, and as it possessed a CARD domain, it was renamed NLRC4 [23]. The CARD domain allows it to directly bind to the CARD of caspase-1 without the participation of ASC [24]. However, NLRC4 is able to bind to ASC and efficiently activate caspase-1, as well as caspase-8, an apoptotic caspase [25].

NLRC5 is a less well-known inflammasome that links both innate and adaptive immune responses by regulating major histocompatibility complex (MHC) I class expression [26]. It is expressed in macrophages, dendritic cells, T cells, B cells and fibroblasts [27]. Moreover, an observed interaction with the NLRP3 inflammasome seemed to have a synergistic effect on IL-1β cleavage, thus it may positively modulate NLRP3 inflammasome activation [28]. Therefore, NLRC5 could form a functional inflammasome, but more studies are needed to know its physiological function more accurately.

Additionally, NOD1 is the founding member of the NLR family, and together with NOD2, they were the first NLRs identified as sensors for PAMPs [29]. NOD1 (NLRC1) and NOD2 (NLRC2) receptors can activate NF-κB and lead the production of inflammatory cytokines. Nevertheless, they have not been described to form an inflammasome complex [30].

## 3. Non-NLR Family Inflammasomes

Recently, other inflammasomes not belonging to the NLR family have been widely described, such as the proteins absent in melanoma 2 (AIM2) and pyrin inflammasomes. AIM2 was described as a sensor able to trigger inflammasome activation, pyroptosis and release of IL-1β and IL-18 in response to intracellularly delivered double-stranded DNA (dsDNA) detection [31]. AIM2 is a member of the ALR family of proteins, composed of an N-terminal PYD domain and a C-terminal HIN (hematopoietic, interferon-inducible and nuclear localization) domain [32]. Moreover, it negatively regulates inflammation and type I interferon (IFN) responses independent of its inflammasome function [33]. Different studies have elucidated a link between increased AIM2 expression and several human diseases, such as atherosclerosis, skin disease or chronic kidney disease [34].

## 4. Mechanisms of NLRP3 Inflammasome Activation

The inflammasome can be understood as a two-sides element and it regulates pathogen infection, but when the immune response triggered is not tightly regulated, it can be involved in pathologies such as CAPS and autoinflammatory disorders [21]. Inflammasomes can recognize a wide variety of endogenous or exogenous, sterile or infectious stimuli within the cell (PAMPs and DAMPs), which trigger its assembly and activation. This process can be explained by considering the upstream sensors recognizing activating signals, the adapters and the downstream effectors [35]. The unfeasibility of a direct interaction between NLRP3 and this diversity of stimuli led to a cellular event producing a conformational change in NLRP3, converting it into an active form. Nevertheless, there is no unique mechanism for the activation of the NLRP3 inflammasome [36]. NLRP3 activation can be triggered by PAMPs and DAMPs detection via PRRs, such as TLRs and NLRs, by cytokine stimulation via IL-1 receptor (IL-1R) or through TNF link to tumor necrosis factor (TNF) receptors TNFR1 and TNFR2 [37]. Moreover, there are mediators that facilitate signal transduction of these receptors: the adaptor protein myeloid differentiation primary response 88 (MyD88), the apoptosis signal-regulating kinase (ASK)1 and ASK2, interleukin 1 receptor-associated kinase (IRAK)1 and IRAK4, caspase-8 (CASP8), Fas-associated protein with death domain (FADD), ubiquitin-binding protein SHARPIN and TRAF-interacting protein with forkhead-associated domain (TIFA). All these elements trigger the transcription of NF-κB, which promotes the transcription of NLRP3 and IL1B genes, habilitating the cell for responding to NLRP3 activators [38].

NLRP3 inflammasome activation in macrophages is a two-step process, thus it requires a priming signal. In the priming process, a non-activating stimulus causes the transcriptional expression of the main components of the inflammasome, this being the ‘first hit’. A second stimuli or ‘second hit’ aggravates the functional activity of the NLRP3 inflammasome [39]. Activation of the NLRP3 inflammasome can be produced by different stimuli, including ionic flux, K+ efflux, Ca2+ influx, Na+ influx and Cl- efflux, reactive oxygen species (ROS) and mitochondrial dysfunction or lysosomal damage. K+ efflux channels P2X purinoceptor 7 (P2X7R) participate in this type of inflammasome activation. Other plasma-membrane-resident Ca2+ channels, namely transient receptor potential melastatin 2 (TRPM2), TRPM7 and transient receptor potential vanilloid 2 (TRPV2), can lead to Ca2+ influx to the cytosol [21] (Figure 1c). Mitochondria regulate homeostasis and respond to changes in intracellular K+ and ROS, resulting in mitochondrial dysfunction and apoptosis. Additionally, mitochondrial apoptotic signaling stimulated by NF-κB cause the production of IL-1β. Oxidized mitochondrial DNA is released as a consequence of mitochondrial dysfunction and apoptosis, and it directly activates the NLRP3 inflammasome [40,41,42].

Apart from the NLR-ASC-caspase-1 canonical inflammasome activation, there is also a non-canonical inflammasome characterized by its activation via caspase-11 in mice with the human orthologs caspase-4/5. Caspase-11 recognizes lipopolysaccharide (LPS) transfected into the cytosol from Gram-negative bacteria, directly binding to its CARD domain. It initiates proteolytic maturation of IL-1β as well as pyroptotic cell death in a GSDMD-dependent manner [35,43]. Non-canonical inflammasome activation by a component of LPS was shown in a previous study where mice lacking caspase-11 were resistant to LPS-induced lethality, even in the presence of TLR4 [44]. However, from the canonical and non-canonical inflammasomes, an alternative pathway of inflammasome activation was observed. It does not require K+ efflux, induction of ASC speck formation, or leading to subsequent pyroptosis, and was spread by TLR4-TRIF-RIPK1-FADD-CASP8 signaling upstream of NLRP3 [45].

NLRP3 inflammasome can be regulated in a post-transcriptional and post-translational level. At the post-transcriptional level, epigenetic factors such as DNA methylation and histone acetylation can regulate NLRP3 mRNA expression in response to Mycobacterium tuberculosis infection [46]. Dysregulation of epigenetic mechanisms could contribute to the pathological development of autoinflammatory syndromes by upregulating the expression of inflammasome components. MicroRNAs are also studied as post-transcriptional regulators of NLRP3 inflammasomes (miR-223, miR-133a, miR-7, miR-30e…) [35,47]. NLRP3 inflammasome activation can also be regulated by post-translational modifications, mainly phosphorylation and ubiquitination. These modifications are often linked. They can provoke different fates on the NLRP3 protein, including the modification of interacting protein networks, trafficking, change in subcellular localization, activation/inhibition of enzymatic activity and proteasomal, lysosomal or autophagic degradation [48]. In fact, a recent study showed that NLRP3 phosphorylation in its LRR domain can regulate inflammasome assembly [49].

## 5. Inflammasome Effector Functions

As previously stated, inflammasomes play a crucial role in the innate immune system by their ability to control the activation of the proteolytic enzyme caspase-1, which leads to proteolytic maturation of the proinflammatory cytokines IL-1β and IL-18, as well as pyroptosis cell death [50]. Mature IL-1β binds to IL-1R, promoting the heterodimerization of the receptor and the subsequent recruitment of components such as MyD88 [51]. IL-1β leads the release of other cytokines such as IL-1α, IL-6 and TNF-α as well as other factors that control growth and differentiation of immune cells [52]. IL-18 participates in many physiological pathways. A higher level of IL-18 can cause metabolic syndromes. For instance, chronic inflammation generated in adipose tissues can lead to insulin resistance and type 2 diabetes mellitus [53].

Another important process carried out by inflammasomes is a lytic form of programmed cell death named pyroptosis. Both canonical inflammasome signaling, recruiting caspase-1, and noncanonical inflammasome, via caspase-4, caspase-5 (in humans) and caspase-11 (in mice), can trigger pyroptosis. It is characterized by cell swelling, membrane lysis, and release of inflammatory compounds into the extracellular space, such as IL-1β, IL-6 and IL-18. Previous studies have shown that gasdermins, a group of pore-forming effector proteins, can play inflammatory caspase-induced pyroptosis, being the N-terminal domain of gasdermin-D sufficient to trigger the process [35,54,55]. Additionally, caspase-8-dependent apoptosis is an additional pathway resulting from inflammasome activation. AIM2 and NLRP3 inflammasomes showed cleaved forms of apical caspase-8 and executioner caspase-3, in response to cytosolic DNA and nigericin, respectively. The process occurred independently from caspase-1 but depended on the inflammasome adapter ASC [56]. Interestingly, a recent study described the capacity of the Z-DNA binding protein 1 (ZBP1), an innate immune sensor capable of activating cell death in the form of pyroptosis, apoptosis and necroptosis (PANoptosis) together with the NLRP3 inflammasome [57].

Whereas the effector functions have been widely studied, there are several additional roles of the inflammasome complexes that have been less characterized. IL-1β is a leaderless cytoplasmic protein whose secretion mechanisms are poorly defined. An endoplasmic reticulum (ER)/Golgi-independent mechanism termed ‘unconventional protein secretion’ was shown, and it was dependent on caspase-1 activation. However, the specific mechanisms and molecular components involved in this process are unclear. Another emerging role of inflammasomes is the activation of eicosanoids, bioactive molecules derived from membrane lipids that play a role in homeostatic and pathological processes. Furthermore, a link between inflammasome activation and autophagy as well as regulation of phagosome maturation have been observed [58].

## 6. The Role of the Inflammasome in Adaptive Immunity and Autoimmunity

The production of proinflammatory cytokines is critical for an effective innate response, as well as a mechanism by which the innate immune system influences the subsequent development of an adaptive immune response [59]. As it is well known, inflammasomes are components of the innate immune system that produce the proinflammatory cytokines IL-1β and IL-18, and they drive the differentiation of specific lineages of helper T cells (Th1, Th2, Th17 and regulatory T cells), which are the main players in adaptive immunity [60]. On the other side, an aberrant inflammasome activation is responsible for the development of CAPS, as well as other common diseases such as metabolic disorders, crystal-related diseases and autoimmune diseases. Inflammation is also crucial in many renal diseases, including acute kidney injury (AKI) and chronic kidney disease (CKD). Although the innate immune system is always involved, in these conditions, the adaptive immunity plays the main role [61].

Concerning autoimmune diseases, they are characterized by self-reactive cells and the overproduction of autoantibodies, produced because of a lack of immunological tolerance and aberrant autoreactive immune responses. The pathogenesis of autoimmune diseases remains to be clarified, but it has been demonstrated that aberration in innate and adaptive immunity is involved. NLRP3 inflammasome has been recently linked with innate immune signal recognition and induction of autoreactive immune responses, probably being a checkpoint in innate immunity to cause distorted adaptive immunity [62,63]. Therefore, how can the NLRP3 inflammasome function affect the development of autoimmune diseases? Cytokines released by the inflammasome, especially IL-1β, produce an inflammatory effect that promotes the development of most autoimmune diseases, including RA and inflammatory bowel disease [64,65]. Furthermore, the NLRP3 inflammasome is also responsible for autoimmune diseases due to an adaptive immune dysfunction. IL-1 mediates T cell proliferation, thus it can promote autoreactive T cells to cause b-cell death [63,66]. NLRP3 inflammasome promotes Th1 differentiation in RA, induced by IL-1β in a caspase-1-dependent manner, and it can also induce differentiation and polarization of Th2, Th17 and dendritic cells in other autoimmune diseases [63,67]. Th17 cells can produce proinflammatory cytokines namely IL-17A, IL-17F, IL-21 and IL-22, while Th1 cells secrete IFN-γ, induced by IL-18, and all of these factors contribute to autoimmunity development [60]. Additionally, autoimmune diseases can be promoted by pyroptosis, leading to the release of cellular debris and its reaction with immune cells, triggering inflammation [68].

Indeed, multiple polymorphisms in inflammasome genes have been associated with the susceptibility and development of autoimmune diseases. For instance, rare gain-of-function variants can be implicated in hereditary inflammatory diseases, characterized by uncontrolled production of IL-1β and/or IL-18, named inflammasomopathies. Mutations in NLRP3 are the prototypic inflammasomopathy, but they have also been described as autoinflammatory diseases associated with mutations that activate the NLRP1, NLRC4 and pyrin inflammasomes [69,70]. Moreover, single nucleotide polymorphisms (SNPs) play a crucial role in autoimmune diseases, and they can affect the priming of inflammasomes, some of their components or end products (IL-1β, IL-18) [71].

## 7. Inflammasome Involvement in Autoimmune Kidney Diseases

A summary of the main publications related to inflammasome investigations in glomerulonephritis is shown in Table 1.

### 7.1. Lupus Nephritis

SLE is a chronic disease that frequently affects the kidney. Lupus nephritis (LN) is the most common renal disease, involving approximately 50% of patients with SLE. This autoimmune disease mostly affects women of the reproductive age. In men, the disorder could be more aggressive. Patients usually have LN at an early age, and it usually presents itself in the initial stages of the disease. Patients with this renal impairment have an increased mortality rate. In total, 10–30% of patients with LN progress to renal failure requiring kidney replacement therapy [72,73].

Irregularities in innate and adaptive immunity contribute to the pathogenesis of SLE. LN occurs when the transcription of genes associated with neutrophils increases. The rise in IFN precedes the activation of neutrophils. The increment of IFN causes the differentiation of B cells into plasmablasts and produces inflammation of specific tissues through neutrophils and active myeloid cells. When these neutrophils die, extracellular neutrophil traps (NETs) appear [72]. NETs are meshing-chromatin fibers combined with granules derived from antimicrobial peptides and enzymes that play an important defense role [74]. These meshes help to maintain antigen-specific autoantibody production [72].

The formation of immune complexes that are deposited in the glomerulus is derived from the production of antibodies against nuclear and cellular antigens. Immune complexes can activate complement and cause kidney damage, especially through the alternative pathway. Plasma interstitial cells generated by B and T cells aggregate in the renal tubulointerstitium also generating the production of autoantibodies [73].

An increase in the inflammasome’s components was observed in biopsies of patients with LN as PYCARD (ASC), caspase-1 and IL-18, indicating their contribution to the disease [75]. Furthermore, the increased transcription of IL-18 in the tubulointerstitial and glomerular compartments [75] correlates with the severity of LN and the onset of proteinuria [76].

#### 7.1.1. In Vitro Model

The activation of the inflammasome in cells of innate immunity could trigger or amplify an autoimmune response. After exposure to an inflammatory stimulus such as LPS, isolated fresh monocytes increase the activation of the inflammasome characterized by the rise in caspase-1, IL-1β and IL-18. A caspase-1 inhibitor added to in vitro cultures reduces IL-18 production [77].

As mentioned previously, the NETosis mechanism contributes to the death of neutrophils in SLE patients. Evidence from groups of researchers suggests that SLE patients are characterized by an imbalance between the development and clearance of NETs, which produces tissue damage [78,79]. Specifically, low-density proinflammatory granulocytes that occur in the bloodstream of SLE patients allow a much greater capacity to produce NETs [79].

Kahlenbertg and her coworkers [80] firstly demonstrated that NETs are, partly, activators of the inflammasome through the externalization of LL-37. NETs externalize various antimicrobial peptides. Specifically, cathelicidin LL-37 is a peptide synthesized by neutrophils, monocytes and macrophages, among others, with activity against several pathogens. LL-37 externalization in NETs has been identified in neutrophils from SLE patients [79]. In this study [80], authors purified and isolated human and murine macrophages. Results showed that LL-37 activated the NLRP3 inflammasome in macrophages and that SLE patients were more likely to activate the inflammasome in response to LL-37 and NETs, compared to macrophages from control patients. This stimulation perpetuates the increase in IL-1β and IL-18, which, in turn, will promote NETosis resulting in disease flares or organ damage, mainly kidneys, skin and brain. Furthermore, their data suggested that the NLRP3 inflammasome is required for caspase-1 activation by LL-37.

#### 7.1.2. Animal Model

It is noteworthy to mention that in various murine studies, NLRP3 has been associated with LN. Kahlenberg et al. [81], studied the role of caspase-1 in the induction of murine lupus. Wild-type mouse models were exposed to pristane developed lupus-related autoantibodies and an active response to INF type I. Following pristane exposure, caspase-1 −/− mice did not have increased levels of IL-1β or IL-18, suggesting that caspase-1 played a role in the transcription of these cytokines. In addition, caspase-1 −/− mice showed less development of autoantibodies and immune complexes related to glomerulonephritis, contrasting with wild-type mice. P2X7, an extracellular ATP-gated ion channel receptor, has also been shown to play a role in NLRP3 activation and LN development. The use of a selective P2X7 antagonist brilliant blue G in MLR/lpr mice produced a downregulation of the NLRP3/ASC/Caspase-1 complex and therefore a suppression of IL-β. This reduced LN severity, proteinuria and blood urea nitrogen levels in mice. Likewise, P2X7/NLRP3 inhibition decreases the Th17:Treg ratio, decreasing anti-double-stranded DNA antibodies(anti-dsDNA). NZM2328 mice that were injected intravenously with adenovirus-expressing interferon-α particles confirmed these results [82]. Increased expression of P2X7 has also been observed in the kidney tissue from patients with SLE [83]. A neutralizing monoclonal antibody to high-mobility box 1 protein, a ubiquitin nuclear protein that binds to DNA, has also been shown to decrease IL-β, IL-6, IL-17 and IL-18 levels and caspase-1 in kidneys of BXSB mice. In addition, this model also attenuated proteinuria, glomerulonephritis, renal immune complex deposits and circulating anti-dsDNA [84].

Another inflammatory pathway that affects LN is NF-κB. NF-κB is a transcription factor that participates in innate and adaptive immunity [85]. In human studies, a correlation has been described between the activation of NF-κB and the histological and renal function impairment [86]. Zhao et al. [87] studied whether the inhibition of both pathways decreased LN progression in lupus-prone MRL / lpr mice. This interest was triggered by the renal pathophysiological role played by NF-κB. Inhibition of NF-κB and NLRP3 by Bay11-7082 prevented their formation and activation, respectively, resulting in an improvement in established kidney damage in LN. Moreover, Bay11-7082 decreased renal immune complex deposits and serum anti-dsDNA levels.

AIM2, has also been implicated in the pathogenesis of SLE. However, its inhibition has been shown to be two-edged in relation to the pathophysiology of LN. Zhang and coworkers analyzed the correlation between the severity of LN and AIM2 in SLE patients and lupus mice. AIM2 expression was elevated in PBMCs from SLE patients. In addition, AIM2 correlated with macrophage activation and was augmented in macrophages induced by lymphocyte-derived apoptotic DNA. The inhibition of AIM2 by siRNA decreased the infiltration of macrophages in renal tissue and produced an improvement in nephritis [88]. However, other researchers found that the inhibition of AIM2 generated susceptibility to developing SLE. p202 negatively regulates AIM2 in some mouse strains, increasing INF and predisposition to SLE [89].

#### 7.1.3. Human Model

The role of the inflammasome in autoimmune diseases have been widely described. Studies with different SNPs related to inflammasome in patients with SLE have been reported in the literature. Pontillo et al. [90] analyzed 14 SNPs in 7 inflammasome genes, such as NLRP1, NLRP3, NLRC4, AIM2, CARD8, CASP1 and IL1B. The study showed, for the first time, an association between SNPs and SLE in a population from southern Brazil. The NLRP1 rs2670660 SNP, especially when combined with the NLRP1 variant rs12150220, confers an increased risk of SLE and developing nephritis, arthritis and rash. Other SNPs also described in autoimmune diseases, such as celiac disease [91] and diabetes [21], were not associated to SLE disease in their population [90]. They also found no association with SLE with respect to AIM2 or IL1B polymorphisms [90]. Furthermore, results from da Cruz et al. found a gain of function in the NLRP3 rs10754558 variant in patients with LN [92].

### 7.2. ANCA Glomerulonephritis

ANCA associated with vasculitis (AAV) is a life-threatening autoimmune disease characterized by an antibody-mediated glomerulonephritis and necrotizing vasculitis. AAV affects small and medium vessels, especially organs such as the kidney and lung. Pauci-immune and necrotizing glomerulonephritis are frequently associated in patients with vasculitis being more prevalent in men over 50 years of age. ANCA vasculitis is usually associated with ANCA-myeloperoxidase (MPO), ANCA-proteinase 3 (PR3) or ANCA-negative serotype positivity. This pathology is classified into different clinical variants such as microscopic polyangiitis, granulomatosis with polyangiitis (Wegener), Eosinophilic granulomatosis with polyangiitis (Churg-Strauss) or vasculitis limited to renal tissue [93]. Both innate and adaptive immunity participate in the development of AAV, although the exact mechanisms remain to be elucidated [93].

Neutrophils play a fundamental role in the pathogenesis of AAV inflicting tissue damage after degranulation induced by ANCA antibodies. Apart from antibodies, T cells are also involved in disease pathogenesis. Neutrophils secrete cytokines that recruit more neutrophils and other inflammatory cells. Infiltration of T cells is also part of the granulomas. The benefit of anti-T cell therapies demonstrates the involvement of this cell in AAV. The Th1 and Th17 phenotypes are involved in the acute phase. An increase in C5a and, therefore, a participation of the alternative complement pathway has been reported [94].

#### 7.2.1. In Vitro Model

Inflammasome components such as cytokines are important mediators in AAV. Both Il-1β and Il-18 have been related to the pathogenesis of AAV, thus the implication of the inflammasome in the inflammation cascade of this disease is expected [61].

Il-18 plays a role in neutrophil chemoattraction independent of TNFα priming [95], contrary to what was reported in other studies [96]. Hewins et al. indicated that, in the presence of anti-TNFα antibody, ANCA-induced superoxide production was not decreased. This would explain the persistence of tissue damage in the presence of anti-TNFα treatment. Furthermore, Hewins and colleagues demonstrated renal Il-18 expression in patients with ANCA vasculitis [95].

#### 7.2.2. Animal Model

Several studies establish ANCA necrotizing crescentic GN (NCGN) in animal models [97]. Dipeptidyl peptidase (DPPI) is a cysteine protease responsible for activating neutrophil serine proteases (NPS) such as cathepsin G (CG), neutrophil elastase (NE) and PR3. These enzymes, responsible for modulating inflammation, have been related to the pathophysiology of ANCA vasculitis. In an anti-MPO antibody-induced experimental model of NCGN, a protective role of DPPI in kidney disease was demonstrated with a local decrease in inflammatory cytokines, especially IL-1β [98]. In fact, the elevation and processing of Il-1β by PR3 and NE has been linked [99]. The group of Scheiber et al. [98] demonstrated that active PR3 or active PR3/NE causes an increase in cytokines and anti-MPO antibodies, generating NCGN. They produced NE-/PR3-mice that were protected from NCGN. This demonstrated the role of NSP in ANCA nephropathy. In addition, they noticed that treatment with anakinra, an IL-1 receptor antagonist, downregulates the inflammatory cascade and protects against NCGN.

Another different pathway that induces Il-1β production and causes NCGN is related to phagocyte NADPH oxidase (Phox). Phox is a heme protein heterodimer from pg91phox and p22phox responsible for generating ROS producing tissue damage [100]. Though, some studies explain that ROS is also involved in the knockdown of inflammation [101]. Another study from the German group of Schreiber et al. [100] discovered the role of Phox in limiting the inflammasome by downregulating its components such as caspase-1 and thus IL-1β. Those authors created an antibody-mediated anti-MPO model. Transplanted mice with gp91phox-deficient or p47phox-deficient bone marrow showed greater histological involvement with more inflammation and necrosis, compared to mice with wild-type bone marrow. Additionally, they also generated pg91phox/caspase-1-deficient bone marrow transplant mice. In this case, mice with double deficiency improved NCGN compared to mice with only pg91phox- deficiency. These results hypothesize that Phox limits the activity of caspase-1 and therefore the role of the inflammasome.

#### 7.2.3. Human Model

Elevated serum levels of the cascade of the inflammasome components have also been found in patients with ANCA vasculitis. IL-18 levels have been seen in patients with ANCA vasculitis regardless of MPO or PR3 values [102]. It has been generally accepted that renal interstitial damage had to be associated with glomerular damage, but there are case reports of patients with ANCA vasculitis with only interstitial injury [103,104]. Tashiro et al. [105] demonstrated no correlation between glomerular damage and interstitial damage in biopsies from patients with ANCA vasculitis. They glimpsed the correlation of IL-1β levels with the severity of tubulointerstitial damage. Moreover, infiltrating macrophages showed positive staining for NLRP3 at the tubulointerstitium without detecting this positivity in the glomerulus. On the contrary, Hewins et al. [95] who found upregulated IL-18 in renal biopsies, reported that positivity in the glomerulus has been found in podocytes while in the tubulointerstitium IL-18-positive has been observed in infiltrating macrophages, myofibroblasts and tubular epithelial cells.

The activation of NOD-like receptors in patients with active stage of ANCA vasculitis is from Wang et al. [106]. The mean optical densities of NOD2, NLRP3 and NLRC5, both in the glomerulus and in the tubulointerstitium, were significantly higher in patients with ANCA vasculitis than in healthy controls, in patients with minimal change disease and in patients with type IV LN. NLRs were mainly expressed in podocytes and in infiltrating monocytes and macrophages, but hardly expressed in glomeruli, results similar to those of Tashiro et al. [105]. The expression of NOD2 and NLRC5 correlated with clinicopathological involvement, while NLRP3 did not [106]. Unlike these researchers, Tashiro and colleagues did correlate NLRP3 with the severity of kidney damage [105].

### 7.3. IgA Nephropathy

IgA nephropathy (IgAN) is the main cause of renal failure due to glomerulonephritis in the world [107]. Components of innate immunity are also involved in this nephropathy. The deposition of IgA aggregates or IgA immune complexes and subsequent activation of T cells causing inflammation is considered the main cause of the disease. The IgA subclass deposited in the glomerulus is the IgA1, which plays the central role in the pathophysiology of the disease. Mesangial cell proliferation is the typical histological finding of IgAN. Mesangial cells undergo proliferation under the action of IL-1, among other cytokines [108]. The contribution of cytokines involved in the inflammasome cascade suggests a role for this inflammatory component in IgAN.

The alternative complement pathway and lectin pathways are also involved in the development of the disease since C3, C4, C4d, properdin, C5b-C9 and mannose binding lectin are usually detected in renal biopsy [109].

#### 7.3.1. Animal Model

Researchers have demonstrated IL-1 expression in kidney tissue affected with IgAN [110,111]. Chen et al. [112], using an animal model of IgAN with ddY mice found decreased mesangial proliferation in mice treated with IL-1 receptor antagonist (IL-1ra). These results suggested that IL-1 is enrolled in IgAN development, evidencing a potential role of the inflammasome cascade in IgAN.

#### 7.3.2. Human/In Vitro Model

The role of NLRP3 in IgAN remains to be discovered. The Canadian team of Chun et al. was the first to evaluate in vivo and in vitro the expression of NLRP3 in the kidney of patients with IgAN and the progression of the disease. They found that NLRP3 was expressed mainly in the tubules with no staining in the normal glomerulus. However, in patients with IgAN, NLRP3 expression was detected in the glomerulus, although it was more increased in the tubules. Both in human kidney biopsies and in low passage human cells, they established a decrease in NLRP3 during tubular damage. Equally the immunostaining results and the NLRP3 mRNA expression corroborated the presence of NLRP3 and its subsequent loss after renal injury. These discoveries suggest that NLRP3 may be a biomarker of tubular integrity. In addition, NLRP3 plays its role in the early stages of kidney disease being implicated in chronic kidney disease. However, due to study limitations, the researchers were unable to report on the functional role of the inflammasome [113].

Other researchers were able to glimpse the role of NLRP3 in the pathophysiology of IgAN. IgA immune complexes elicited the activation of the NLRP3 inflammasome in macrophages. This, in turn, stimulated the production of ROS by the mitochondria. They performed a mouse model with IgAN knockout for NLRP3. The generation of IgA immune complexes was inhibited by knockout mice. A regaining in renal function was described in NLRP3 knockout and in the kidney-targeting delivery of shRNA of NLRP3. Finally, the researchers clarify that they cannot exclude the role of other inflammasomes in IgAN and that the use of shNLRP3 could be a treatment to improve or prevent the disease [114].

### 7.4. Anti-Glomerular Basement Membrane Glomerulonephritis

Anti-glomerular basement membrane (anti-GBM) is an infrequent autoimmune disease that affects the small vessels of the kidneys and lungs. Patients develop antibodies against the non-collagenous domain of the α3 chain of type IV collagen present in the basal membrane of those organs [115]. Although the humoral immunity plays a central role, the participation of cellular immunity has also been reported. Thus, the IgG1 and IgG3 subclasses have been clearly related to the severity of the disease. The deposition of antibodies in kidney vessels can origin inflammation by activating complement and the Fc receptor. On the other hand, the increase in CD4+ T cells has been correlated with the severity of the disease. Peripheral CD4+ progress in the presence of α3 (IV) NC1. In addition, in animal models, CD4+ has been shown to be a trigger for the development of anti-GBM antibodies [116].

#### Animal Model

The major cytokines derived from the inflammasome cascade, IL-1 and IL-18, have been shown to have a pathophysiological role in patients with anti-GBM disease. In a mouse model of anti-GBM, Il-1β −/− and IL1 type 1 receptor (IL-1R) −/− mice, the role of IL-1 isoforms, IL-1α and IL-1β, in anti-GMB GN was studied. IL-1β mice showed less development of crescentic formations, recruitment of macrophages and T cells, while IL-1R1 −/− mice appeared to have a role in the immune response, since they had fewer antibodies in serum [117]. Furthermore, other animal models have demonstrated the proinflammatory role of IL-18 in renal inflammation [118].

Glomerular infiltration by macrophages is probably one of the major sources of IL-1 cytokine production. Several studies have analyzed the chemoattractant role of this cytokine and have implemented treatment with antagonists IL-1ra in a rat model with anti-GBM. Both the group of Lan et al. [119] and Tang et al. [120], demonstrated that by using IL-1ra there was a decrease in the infiltration of glomerular macrophages and an improvement in proteinuria. Lan et al. [119] also revealed a stoppage of renal function worsening and prevented histological progression such as the formation of glomerular crescents. Tang et al. [120] obtained a decreased expression of ICAM-1 after treatment with IL-1ra, which was also associated with a decline in the infiltration of polymorphonuclear (PMN) cells and monocytes.

However, following the findings found by Timoshanko et al. [117] on the contribution of IL-1β in nephritis in anti-GBM, other authors concluded, using a murine model, that only dendritic cells that reside mainly in the tubulointerstitium express pro-IL-1β and therefore they activate NLRP3 and caspase-1 secreting mature IL-1β. They showed that the inflammasome axis does not contribute to glomerular inflammation since glomerular cells could not produce IL-1β during sterile inflammation [121].

## 8. Final Remarks

As comprehensively detailed, many studies have demonstrated the role of inflammasome in glomerulonephritis. However, further insights into the pathophysiological mechanism research focus on inflammasome and autoimmune diseases are needed.

On the other hand, the participation of the inflammasome in immunity encourages the need for new therapeutic weapons aimed at its modulation. Recently, antagonists of IL-1ra are already approved to treat non-renal diseases such as rheumatoid arthritis, CAPS, Familial Mediterranean fever and Still’s disease. Additionally, for now there is development for treating autoimmune diseases in patients that are non-responsive or over time are refractory to treatment with TNF-α antagonists and/or T-cell co-stimulation antagonists with an IL-18 antagonist. Given that the IL-1 blockade or IL-18 antagonist have been successful in non-human models of renal diseases modulating inflammasome activation. Perhaps, we are ready to introduce these targets in the nephrological clinics. However, the blockade of a single cytokine could be not enough to downregulate the activation of the inflammasome, then polytherapy could be considered. To our knowledge, while there are developed clinical trials about autoimmune and inflammatory diseases, there are no ongoing clinical trials involving glomerulonephritis and inflammasomes. Thus, further efforts in the exploration into how treatments affect the activity of the inflammasome axis could be a promising therapy.

## Figures and Tables

**Table 1:** Summary table: studies about the inflammasome’s role in glomerulonephritis.

**LUPUS NEPRHITIS**	
**Significant Findings**	**References**
**IN VITRO MODEL**	
**LL-37:**	
LDGs have the capacity to produce NETs which increase the externalization of immunostimulatory proteins and autoantigens as LL-37, IL-17 and dsDNA. Kidneys from SLE patients are infiltrated by netting neutrophils which show LL-37 and dsDNA explaining the role of aberrant lupus neutrophils the pathogenic role of NETs.	Villanueva et al., Journal of Immunology, 2011.
NLRP3 is activated by NETs and the expression of the NETs-associated protein LL-37. This stimulus contributes to the production of IL1β and IL-18 causing NETosis.	Kahlenberg et al., Journal of immunology, 2013.
**Expression of axis’s inflammasome:**	
Isolated fresh monocytes from SLE patients increased inflammasome activation described by the elevated expression of Caspase-1, IL-1β and IL-18.	Perez-Alamino et al., Reumatologia Clinica, 2021.
**ANIMAL MODEL**	
**P2X_7:_**	
Increased expression of P2X_7_ has been observed in kidney biopsies from patients with SLE.	TTurner et al., Nephrology, dialysis, transplantation, 2007.
Upregulation of P2X_7_/NLRP3 in kidneys of MRL/lpr mice associates an increase in IL-1β and renal damage developing LN.P2X_7_ inhibition decreases autoantibodies and immune complexes deposited in the kidneys.	Zhao et al., Arthritis Rheumatology, 2013.
**NFκB and NLPR3:**	
The inhibition of NFκB and NLPR3 by Bay11-7082 in MRL/lpr mice reduces nephritis, the levels of IL-1β, TNF-α and anti-dsDNA and the deposition of immune complexes.	Zhao et al., International Immunopharmacology, 2013.
**AIM2:**	
AIM2 is augmented in macrophages induced by lymphocyte-derived apoptotic DNA. Its knock-down by siRNA ameliorates infiltration of macrophages in tissues.	Zhang et al., Journal of Clinical Immunology, 2013.
p202 limits AIM2. This increases INF causing susceptibility to murine lupus.	Yin et al., Cell reports, 2014.
**Caspasa-1:**	
The caspase-1 −/− mouse model exposed to pristane protected against the development of autoantibodies related to SLE, nephritis and the action of type I INF.	Kahlenberg et al., Arthritis and Rheumatology, 2014.
**HMGB1:**	
Blocking HMGB1 in BXSB mice reduces the machinery of NLPR3 and improves renal inflammation.	Zhang et al., Scandinavian Journal of immunology, 2014.
**HUMAN MODEL**	
**NLRP1:**	
The NLRP1 rs2670660 and NLRP1 rs12150220-rs2670660 A-G haplotype polymorphisms were associated with SLE and the event of nephritis, arthritis and rash.	Pontillo et al., Autoimmunity, 2012.
**NLPR3, NLRP1, Caspasa-1, AIM2:**	
The variant rs10754558 NLRP3 was more common in SLE patients with nephritis. The stimulus with LPS+ATP generated the expression of NLRP1, AIM2, CASP1 and IL1β genes, indicating that NLRP1 is responsible for the IL-β production reflected in monocytes.	da Cruz et al., Immunogenetics, 2020.
**ANCA GLOMERULONEPHRITIS**	
**Significant Findings**	**References**
**IN VITRO MODEL**	
**IL-18:**	
Il-18 expression is upregulated in patients with ANCA vasculitis.	Hewins et al., Kidney International, 2006.
**ANIMAL MODEL**	
**NPS:**	
NE^-^/PR3^-^ mice in anti-MPO antibody-induced model reduce local cytokines and induction of NCGN.	Schreiber et al., Journal of the American Society of Nephrology, 2012.
**NADPH oxidase:**	
An antibody-mediated anti-MPO model, gp91^phox^-deficient or p47^phox^-deficient mice had worsening NCGN. Gp91^phox^-deficient/caspase-1 double-deficient mice improved NCGN, suggesting that Phox limits the activity of caspase-1 and thus of the inflammasome.	Schreiber et al., Journal of the American Nephrology, 2015.
**HUMAN MODEL**	
**IL-18:**	
IL-18 is elevated in the serum from patients diagnosed with ANCA vasculitis compared to healthy controls. The increase in IL-18 is regardless of MPO/PR3 levels.	Hultren et al., Autoimmunity, 2007.
**NLRP3, NOD2, NLRC5:**	
The investigators glimpsed the role of NLRP3 in the tubulointerstitial compartment and the correlation of IL-1β levels with the severity of tubulointerstitial injury in the glomerulus.	Tashiro et al., Clinical Nephrology, 2016.
NOD2, NLRP3 and NLRC5 were mostly expressed in podocytes and in infiltrating monocytes and macrophages, but barely expressed in glomeruli.	Wang et al., Journal of Translational Medicine, 2019.
**IGA NEPHROPATHY**	
**Significant Findings**	**References**
**ANIMAL MODEL**	
**IL-1ra:**	
The use of Il-1 receptor antagonist in a IgAN’s mouse model (ddY mice) ceases the exacerbation of the disease.	Chen et al., American Journal of Kidney Diseases, 1997.
**HUMAN/IN VITRO MODEL**	
NLRP3 was mostly expressed in the tubules with no staining in the glomerulus of normal kidneys. Nevertheless, in patients with IgAN, NLRP3 expression was detected in the glomerulus, though it was more increased in the tubules. In human kidney biopsies and in low passage human cells, they established that NLRP3 was decreased during tubular damage. Equally the immunostaining results and the NLRP3 mRNA expression confirmed the presence of NLRP3 and its subsequent loss after renal injury.	Chun et al., Scientific reports, 2016.
IgAN knockout NLRP3 mice model was generated. The production of IgA immune complexes was inhibited by knockout mice. NLRP3 knockout mice and the kidney-targeting delivery of shRNA of NLRP3 improve renal function.	Tsai et al., scientific reports, 2017.
**ANTIGLOMERULAR BASEMENT MEMBRANE GLOMERULONEPHRITIS**	
**Significant Findings**	**References**
**ANIMAL MODEL**	
**IL-1ra:**	
IL-1ra protects against clinical and histological worsening in a rat anti-GBM model.	Lan et al., Kidney International, 1993.
In a rat anti-GBM model, IL-1ra diminishs proteinuria and the expression of adhesion molecules of PMN, such as ICAM-1.	Tang et al., The Journal of Clinical Investigation, 1994.
**IL-18 and IL-12p40:**	
Anti-GBM mice model with IL-12p40−/−, IL-18−/− and both IL-12p40−/− and IL-18 demonstrate IL-12p40 as a crucial cytokine chain in nephritogenic Th1 responses and IL-18 as a proinflammatory local (renal) cytokine.	Kitching et al., Journal of the American Society of Nephrology, 2005.
**IL-1β** **and IL-1RI:**	
An anti-GBM IL-1β −/− and IL-1RI −/− mouse model was formed. IL-1β −/− mice demonstrated a reduction in crescent formation and cell recruitment. IL-1RI −/− mice presented less serum titers antibodies, less proteinuria and reduced serum creatinine.	Timoshanko et al., Journal of the American Society of Nephrology, 2004.
Anti-GBM nephritis develops independently of the NLRP3-caspase-1 axis due to the inability of glomerular cells to generate IL-1β.	Lichtnekert et al., Plos One, 2011.

LDG, low density granulocytes; NETs, neutrophils extracellular traps; dsDNA, anti-double-stranded DNA; SLE, systemic lupus erythematosus; LN, lupus nephritis; NFκB, nuclear factor kappa B; TNF-α, tumor necrosis factor α; siRNA, small interfering RNA; INF, interferon; HMGB1, high-mobility group box 1 protein; LPS, Lipopolysaccharide; ATP, adenosine triphosphate; CASP1, caspase-1; ANCA, Anti-Neutrophilic Cytoplasmic Autoantibody; NE, neutrophil elastase; PR3, proteinase 3; MPO, myeloperoxidase; NCGN, necrotizing crescentic glomerulonephritis; Phox, NADPH oxidase; PR3, proteinase 3; IgAN, IgA nephropathy; mRNA, messenger RNA; shRNA, short hairpin RNA; Il-1ra, interleukin-1 receptor antagonist; anti-GBM, anti-glomerular basement membrane; PMN, polymorphonuclear; ICAM-1, intercellular adhesion molecule-1; IL-1RI, IL1 type 1 receptor.

## Data Availability

Not applicable.
